# Comparative transcriptomic and rhizosphere microbiome profiling and cross-omics association analysis of wild and cultivated *Panax japonicus* var. *major*

**DOI:** 10.3389/fpls.2026.1961578

**Published:** 2026-10-05

**Authors:** Qiuyue Xiang, Yunqing Yang, Xiang Gao, Han Zhang, Ruihong Gui, Kesong Li, Yaqiong Li

**Affiliations:** School of Chinese Materia Medica, Yunnan University of Chinese Medicine, Kunming, Yunnan, China

**Keywords:** domestication, Panax japonicus var. major, rhizosphere microbiome, terpenoid biosynthesis, transcriptome, WGCNA

## Abstract

**Introduction:**

*Panax japonicus* var. *major* is an important medicinal plant endemic to China and is rich in terpenoid saponins with diverse pharmacological activities. Excessive harvesting of wild resources has accelerated the transition toward cultivation, raising questions about differences between wild and cultivated populations.

**Methods:**

Transcriptomic and rhizosphere microbiome analyses were conducted in sampled wild and cultivated populations, followed by an exploratory cross-omics association analysis.

**Results:**

Transcriptome comparisons identified 10,416, 8,782, and 5,441 differentially expressed unigenes in roots, rhizomes, and leaves, respectively. Functional enrichment analyses showed differences in plant hormone signal transduction, MAPK signaling, plant–pathogen interaction, phenylpropanoid biosynthesis, and terpenoid biosynthesis pathways. Several terpenoid biosynthesis-related genes, including AACT, HMGS, HMGR, DXS, and TPS, had higher transcript levels in wild plants. Rhizosphere microbiome analyses showed differences in bacterial and fungal community composition among the sampled groups. Exploratory cross-omics analysis identified candidate associations between several microbial taxa and terpenoid biosynthesis-related genes.

**Discussion:**

These taxonomic assignments do not establish the functional roles of the detected microorganisms. Because terpenoid and saponin concentrations were not measured, the transcriptomic differences do not demonstrate greater metabolite accumulation or improved medicinal quality. Moreover, because the populations were sampled from different sites and the microbiome data lacked independent biological replication within each sampling group, the observed patterns do not establish effects of cultivation status or habitat, nor do they demonstrate causal plant–microbe interactions. The results provide hypotheses for future controlled studies of *P. japonicus* var. *major*.

## 1 Introduction

Within the genus Panax, *P. ginseng* has a long history of medicinal and dietary use and contains diverse bioactive constituents, including ginsenosides, polysaccharides, peptides, and volatile compounds (Zhang et al., 2025). *Panax japonicus* C.A. Mey. var. *major* (Burk.) C.Y. Wu et K.M. Feng (Araliaceae) is an important medicinal plant used in traditional Chinese medicine (Chinese Pharmacopoeia Commission, 2025) (**Figure 1**). Known locally as Zhuzishen, Yesanqi, and Gedaqi, its dried rhizome has long been used by the Naxi and Yi ethnic groups (Li et al., 2023). Reported constituents include diverse saponins (Chen et al., 2019; Zhang et al., 2021; An et al., 2023), and pharmacological studies have examined cardiovascular, hemostatic, antitumor, and tonic-related activities of its extracts or constituents (He et al., 2018; Li et al., 2020b; Pang et al., 2024; 2025).

**Figure 1:**
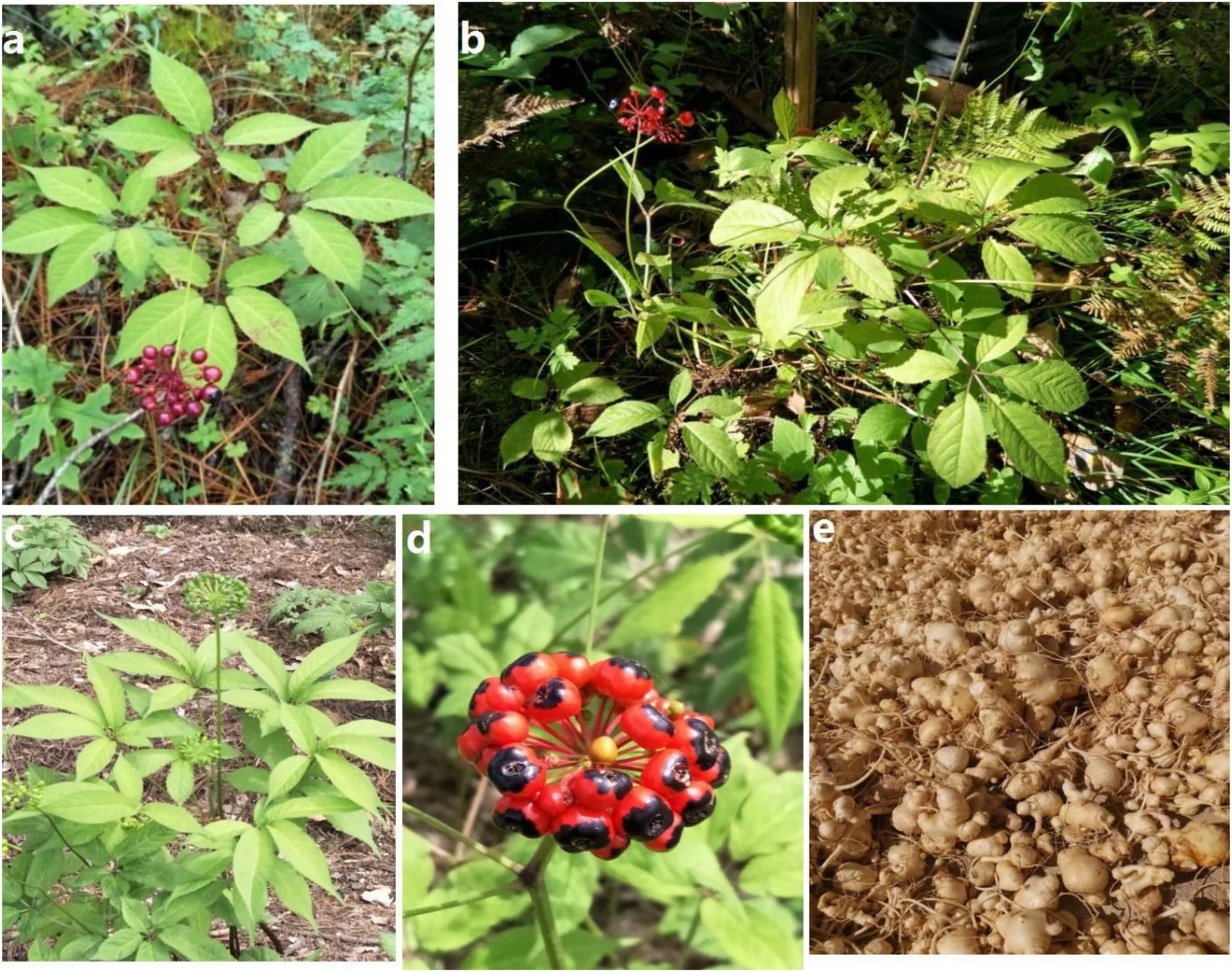
*Panax japonicus* var. *major* **(a, b)** in the wild; **(c, d)** in a cultivated environment; and **(e)** rhizomes and roots.

Endemic to China, *P. japonicus* var. *major* occurs in parts of Yunnan and Shaanxi Province (Liu et al., 2021). Demand and poorly managed harvesting have contributed to declines in wild populations (Yang et al., 2016). Consequently, *P. japonicus* var. *major* has been listed as a national second-class protected wild plant in China (National Forestry and Grassland Administration and Ministry of Agriculture and Rural Affairs, 2021).

As wild resources have declined, cultivated material has become increasingly important for medicinal use. Both wild and cultivated plants may experience drought, low temperature, and other stresses; the two categories should therefore not be contrasted as ‘stressed’ and ‘unstressed.’ Nevertheless, in this study they were sampled under different environmental and management conditions, which may have contributed to the observed transcriptional and rhizosphere microbial differences. Plant specialized metabolites participate in environmental responses and in interactions with associated microorganisms (Singh et al., 2023).

Plant roots release a variety of organic compounds as root exudates, including sugars, amino acids, organic acids, and enzymes. These exudates can attract and recruit beneficial microorganisms into the rhizosphere, leading to the formation of complex microbial communities (Enagbonma et al., 2023). Moreover, beyond acting as signaling molecules to promote communication between plants and microorganisms, root exudates also regulate the growth, metabolism, and function of microorganisms. Recent studies have shown that metabolites in root exudates, such as triterpenes and flavonoids, are essential for plants to assemble a functional microbiome, and that changes in plant genetics caused by cultivation programs lead to different root exudate compositions, thereby disrupting the assembly and function of the microbiome (Nerva et al., 2022; Vandenkoornhuyse et al., 2015).

Previous studies have reported associations between cultivation practices and changes in plant-associated microbiota, potentially linked to variation in root architecture, root exudation, and defense responses (Martínez-Romero et al., 2020). Plant–microbe interactions can influence plant growth, development, disease resistance, and metabolic processes, while beneficial plant-associated microorganisms may contribute to plant growth and health (Nerva et al., 2022; Herms et al., 2022). These observations provide a basis for examining whether wild and cultivated plants differ in disease resistance, growth, and medicinally relevant constituent profiles. Therefore, comparing transcriptomic and rhizosphere microbial profiles of wild and cultivated *P. japonicus* var. *major* may help identify patterns associated with their contrasting habitats.

This study compared transcriptomic profiles and rhizosphere microbial communities among sampled wild and cultivated *P. japonicus* var. *major* populations. The objectives were to identify tissue-specific transcriptional differences and describe rhizosphere community patterns. The analysis was not designed to establish a plant-microbiome regulatory network.

## 2 Materials and methods

### 2.1 Plant and soil materials

Wild *P. japonicus* var. *major* specimens were collected from Lameirong village, Ludian town, Yulong County, Lijiang City, Yunnan Province (27° 22′ 18″ N, 99° 50′ 21″ E) (group WI, **Table 1**), and cultivated samples were collected from Ludian village, Ludian town, Yulong County (27° 24′ 08″ N, 99° 52′ 36″ E; group CP, **Table 1**). Representative wild and cultivated plants and the sampled underground tissues are shown in **Figure 1**. Both WI and CP were collected in August 2024 from 3-year-old plants, and the leaves, rhizomes, and roots were extracted for total RNA analysis. For the sequencing of rhizosphere microorganisms, additional soil samples of wild (group WI1: 27° 23′ 08″ N, 99° 52′ 36″ E) and cultivated *P. japonicus* var. *major* (group CP1: 27° 23′ 36″ N, 99° 52′ 28″ E) were collected. The 16S rRNA and ITS sequencing was carried out on rhizosphere soil samples from wild (WI, WI1) and cultivated (CP, CP1) plants. The identity of the plants was verified by Prof. Ya-qiong Li (**Table 1**). Two sampling locations were included for each population type in the rhizosphere microbiome analysis to reduce reliance on a single location and to provide broader representation of the sampled wild and cultivated habitats. The additional locations were intended to capture part of the spatial variation in rhizosphere microbial communities within each population type. However, because the wild and cultivated locations differed in environmental conditions, they were not regarded as controlled replicates for testing the effect of domestication. Transcriptomic analysis was conducted using plant tissues collected from the WI and CP locations, whereas rhizosphere microbiome analysis included samples from WI, WI1, CP, and CP1.

**Table 1:** Collection locations of wild and cultivated *Panax japonicus* var. *major*.

Sample	Collection location	Latitude and longitude	Sample collection date
WI	Lameirong village, Ludian town Yulong County, Lijiang City, Yunnan Province	27° 22′ 18″ 99° 50′ 21″	2024.8.8
CP	Ludian village, Ludian town Yulong County, Lijiang City, Yunnan Province	27°24′ 08″ 99°52′36″	2024.8.8
WI1	Lameirong village, Ludian town Lameirong County, Lijiang City, Yunnan Province	27°23′08″ 99°52′36″	2024.8.8
CP1	Ludian village, Lutou town Yulong County, Lijiang City, Yunnan Province	27°23′36″ 99°52′28″	2024.8.8

The wild and cultivated samples were collected from separate sites and were therefore not assumed to experience equivalent growth conditions. Cultivated plants were grown in an open sandy-loam field beneath black shade netting that reduced incident light by 60%, whereas wild plants occurred in unmanaged mountainous vegetation. Soil physicochemical properties, site altitude, vegetation composition, previous field management, fertilizer and pesticide application records, and site-level microclimatic variables were not measured or available for this sampling campaign. These unmeasured variables may have contributed to the observed transcriptomic and rhizosphere microbial differences and prevent attribution of those differences to cultivation status alone.

*Panax japonicus* var. *major* (Zhuzishen) is listed as a national second-class protected wild plant in China (National Forestry and Grassland Administration and Ministry of Agriculture and Rural Affairs, 2021). Six wild and eight cultivated plants were collected. For transcriptome analysis, three independent plants were analyzed for each tissue–population combination. The archived transcriptome report (project MJ20240911253) comprised 18 libraries: A1–A3, B1–B3, and C1–C3 represented wild roots, wild rhizomes, and wild leaves, respectively, whereas E1–E3, F1–F3, and G1–G3 represented cultivated roots, cultivated rhizomes, and cultivated leaves, respectively. For microbiome analysis, one rhizosphere soil sample was obtained from each sampling group (WI, WI1, CP, and CP1), and each soil sample was divided into three technical sequencing aliquots. These aliquots were not treated as independent biological replicates.

### 2.2 Transcriptomic sequencing

Roots, rhizomes, and leaves were collected separately from three 3-year-old wild plants and three 3-year-old cultivated Panax japonicus var. major plants. Each plant served as an independent biological replicate, resulting in three biological replicates for each tissue–population combination and 18 RNA-seq libraries in total. The rhizome is the principal medicinal part, whereas the leaf was included as a non-medicinal photosynthetic tissue to determine whether transcriptional differences between the sampled wild and cultivated plants were restricted to the medicinal organ or were also detectable in an aboveground tissue.

Total RNA was extracted using TRIzol reagent (Invitrogen, Carlsbad, CA, USA) according to the manufacturer’s instructions. RNA concentration, purity, and integrity were evaluated by spectrophotometry, agarose gel electrophoresis, and microfluidic electrophoresis before library preparation. Qualified total RNA was used for poly(A) mRNA enrichment with oligo(dT)-attached magnetic beads. The enriched mRNA was fragmented into approximately 300-bp fragments, followed by first- and second-strand cDNA synthesis, end repair, 3′-adenylation, adaptor ligation, fragment selection, PCR enrichment, and library purification. The 18 libraries were sequenced on an Illumina NovaSeq platform using 150-bp paired-end sequencing by Shanghai Majorbio Bio-pharm Technology Co., Ltd. (Shanghai, China).

Adaptor-containing reads, reads containing undetermined bases, and low-quality reads were removed from the raw data. The quality of the clean reads was evaluated based on the sequencing error rate, Q20, Q30, and GC content. Clean reads from all libraries were assembled *de novo* using Trinity. The initial assembly was optimized using TransRate and CD-HIT to remove low-quality and redundant sequences. The longest transcript within each Trinity cluster was retained as the representative unigene, and assembly completeness was assessed using BUSCO (Simão et al., 2015).

The assembled unigenes were functionally annotated against the NCBI non-redundant protein, Swiss-Prot, Pfam, EggNOG, Gene Ontology, and Kyoto Encyclopedia of Genes and Genomes databases. Unigene abundance was quantified using RSEM and expressed as transcripts per million. Differential-expression analysis between the sampled wild and cultivated plants was conducted separately for roots, rhizomes, and leaves using DESeq2 (Love et al., 2014). Unigenes with |log_2_(fold change)| ≥ 1 and a Benjamini–Hochberg-adjusted P value < 0.05 were considered differentially expressed. GO and KEGG enrichment analyses were performed using the differentially expressed unigene sets, and terms or pathways with an adjusted P value < 0.05 were considered significantly enriched.

### 2.3 Microbiome analysis

The topsoil (10–15 cm) was removed, and intact root systems of cultivated and wild *P. japonicus* var. *major* were excavated from a depth of approximately 20 cm using a sterile shovel. Loosely adhering soil was removed, and the soil that remained attached to the roots within an approximately 1–2 mm layer was collected with a sterile brush and operationally defined as rhizosphere soil. The collected soil was passed through a 2-mm sieve and transferred to sterile tubes. Only this rhizosphere soil fraction was used for microbial DNA extraction and amplicon sequencing; root-surface (rhizoplane) and root-interior (endosphere) samples were not collected or sequenced.

Total microbial DNA was extracted from each rhizosphere soil sample using the CTAB method, and DNA integrity was examined by 1% agarose gel electrophoresis. The bacterial 16S rRNA V3–V4 region was amplified using 338F (5′-ACTCCTACGGGAGGCAGCAG-3′) and 806R (5′-GGACTACHVGGGTWTCTAAT-3′), whereas the fungal ITS1 region was amplified using ITS1F (5′-CTTGGTCATTTAGAGGAAGTAA-3′) and ITS2R (5′-GCTGCGTTCTTCATCGATGC-3′). Sample-specific barcodes were added to the primers. PCR was performed using TransStart FastPfu DNA Polymerase (TransGen AP221-02) on an ABI GeneAmp 9700 thermal cycler. Three parallel PCR reactions were performed for each soil sample and pooled within the sample. Amplicons were examined by 2% agarose gel electrophoresis, purified using an AxyPrep DNA Gel Extraction Kit, quantified using a QuantiFluor-ST fluorometer, and used to prepare target-specific libraries with a TruSeq DNA Sample Prep Kit. The libraries were sequenced in paired-end mode on an Illumina NextSeq 2000 platform under project MJ20240911358. The archived report did not retain a nominal PE150/PE250/PE300 designation or a flow-cell identifier; therefore, the strategy is reported as paired-end sequencing. No dedicated extraction blank, PCR-negative control, positive control, or mock-community dataset was documented or used for downstream correction.

Paired-end reads were merged according to their overlapping regions and demultiplexed using sample-specific barcodes. Identical optimized sequences were dereplicated, singleton sequences were removed, and OTUs were clustered at 97% sequence similarity using UPARSE v7.0.1090. Chimeric sequences were removed during clustering, and all optimized sequences were mapped to representative OTU sequences at ≥97% similarity to construct the OTU abundance table. Representative bacterial and fungal OTUs were classified using the RDP Classifier v2.11 with a confidence threshold of 0.70 against SILVA Release 138 (Quast et al., 2013) and UNITE Release 8.0, respectively. OTUs assigned to non-target lineages, including chloroplast and mitochondrial sequences in the bacterial dataset, were excluded before downstream analysis. Rarefaction curves were generated by random subsampling to assess sequencing sufficiency. For diversity comparisons, the bacterial and fungal OTU tables were rarefied separately to the minimum retained sequencing depth within each dataset; the fungal table was rarefied to 47,727 sequences per aliquot. Taxonomic composition was expressed as relative abundance within each aliquot.

Principal coordinate analysis (PCoA) was performed on Bray–Curtis dissimilarities calculated from the rarefied OTU tables to visualize beta diversity patterns among the four individual soil samples (**Figure 2**). This distance-based approach has been widely used in rhizosphere microbiome studies of Panax species (Fang et al., 2022). Because only one independent rhizosphere soil sample was collected per sampling group (WI, WI1, CP, and CP1), the three technical sequencing aliquots from each sample were not treated as biological replicates. Consequently, no group-level inferential statistics (e.g., PERMANOVA, Kruskal–Wallis, ANOVA, or LEfSe) were performed on the microbiome data. All alpha diversity indices, PCoA ordinations, and taxonomic relative-abundance patterns are presented descriptively for the four individual samples collected.

**Figure 2:**
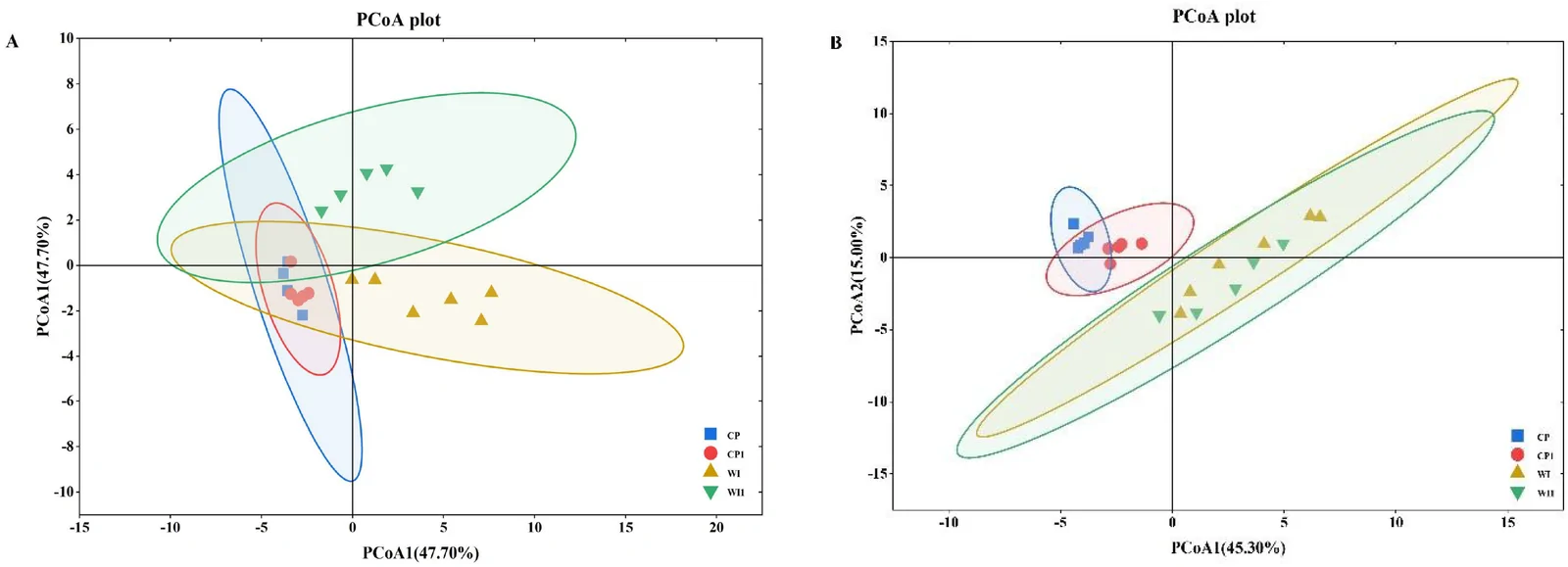
Analysis of OTUs in samples of *Panax japonicus* var. *major*: **(A)** principal coordinate analysis (PCoA) of bacterial communities; **(B)** PCoA of fungal communities.

### 2.4 Co-occurrence network analysis

The weighted gene co-expression network analysis (WGCNA) package in R was used to explore candidate associations between gene-expression profiles and microbial operational taxonomic unit (OTU) data. An adjacency matrix was constructed using a soft-thresholding power of 12 and transformed into a topological overlap matrix using the TOM similarity algorithm. Because only three biological replicates were available per transcriptomic group and the microbiome data were based on technical replicates of one soil sample per sampling group, the network analysis was treated as exploratory. The resulting modules and edges were interpreted as candidate associations rather than stable co-expression modules or regulatory relationships, and no claims of module robustness or preservation were made.

### 2.5 Validation of RNA-seq expression profiles by qRT-PCR

RNA-seq expression patterns were validated by reverse-transcription quantitative PCR (qRT-PCR). Total RNA from three independent biological replicates per group was reverse-transcribed into cDNA, and each biological replicate was analyzed in three technical PCR replicates. Sixteen genes involved in terpenoid biosynthesis, including AACT, HMGS, HMGR, DXS, DXR, HDS, HDR, FDS, GDS, TPS, MVK, PMK, MVD, IDI, MCT, and CMK, were selected for qRT-PCR validation. qRT-PCR was performed using TB Green Premix Ex Taq II on a real-time PCR system, with β-actin as the reference gene. Relative expression was calculated using the 2−ΔΔCt method. The mean amplification efficiency of the qRT-PCR primer pairs was 91%. Differences between wild and cultivated samples were evaluated using two-tailed Student’s t-tests based on the three biological replicates, with *P* < 0.05 considered significant. Technical replicates were averaged before statistical analysis.

## 3 Results

### 3.1 Overview of transcriptome and rhizosphere microbiome sequencing data

Transcriptomes were generated from roots, rhizomes, and leaves of sampled 3-year-old wild and cultivated *P. japonicus* var. *major* plants (**Figure 3**). Rhizosphere soil, rather than root tissue, was used for bacterial 16S rRNA and fungal ITS sequencing. The transcriptome data were assembled and annotated to compare tissue-specific expression patterns between the sampled populations.

**Figure 3:**
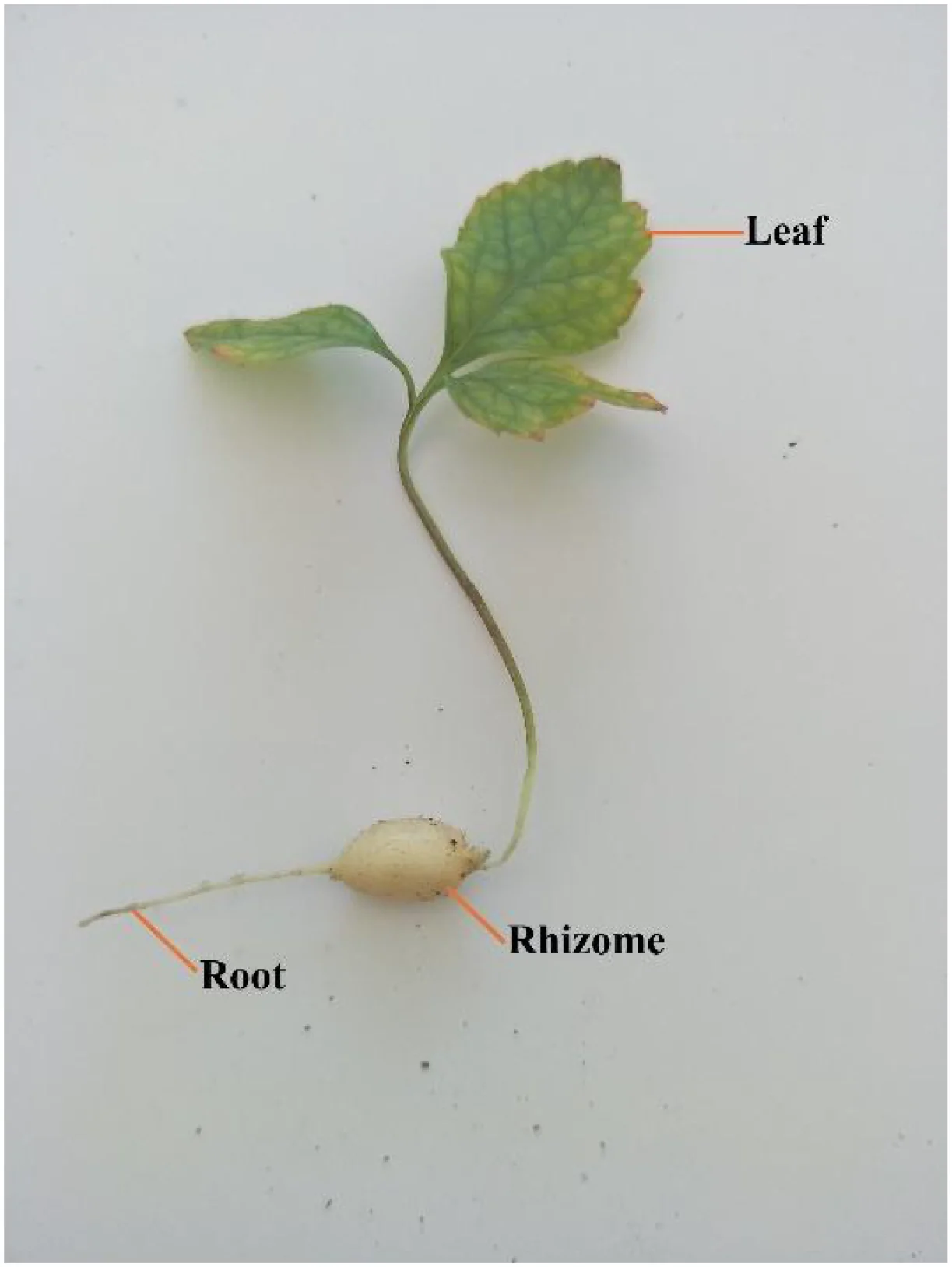
*Panax japonicus* var. *major* leaf, rhizome, and root of a 3-year-old seedling for transcriptome sequencing.

Sequencing of the 18 transcriptome libraries generated 787,951,436 raw reads. After quality filtering, 782,454,756 clean reads comprising 117,063,982,867 bases (117.06 Gb) were retained. Each library yielded at least 5.93 Gb of clean data, with Q30 values of 96.10%–96.42% and GC contents of 42.45%–46.44%. *De novo* assembly generated 157,226 unigenes and 266,108 transcripts. The assembled unigenes had a total length of 134,739,011 bp, a mean length of 856.98 bp, and an N50 of 1,205 bp; 156,609 unigenes were detected as expressed. For the fungal ITS dataset, D_1–D_3, H_1–H_3, I_1–I_3, and J_1–J_3 corresponded to WI, WI1, CP, and CP1, respectively. A total of 638,482 optimized ITS1 sequences were retained after paired-end merging and quality filtering. The retained sequence counts were 52,157, 58,323, and 48,009 for WI; 51,646, 55,125, and 50,812 for WI1; 53,701, 59,083, and 53,977 for CP; and 54,265, 47,727, and 53,657 for CP1. Per-aliquot depth ranged from 47,727 to 59,083 sequences, and the mean merged sequence length was 239.75 bp (range 52–538 bp). The rarefaction curves approached a plateau, indicating that the sequencing depth captured most detectable fungal OTUs. Corresponding per-aliquot bacterial sequencing statistics are provided in **Supplementary Table S3**.

### 3.2 Comparative transcriptomics data between wild and cultivated *Panax japonicus* var. major

Unigene expression levels were quantified using RSEM, and differential-expression analysis was performed separately for roots, rhizomes, and leaves using DESeq2. Of the 157,226 assembled unigenes, 156,609 were detected as expressed, and 87,171 received at least one functional annotation (**Supplementary Table S6**). The comparisons identified 10,416 differentially expressed unigenes in roots, including 8,932 with higher and 1,484 with lower expression in wild plants; 8,782 in rhizomes, including 4,822 with higher and 3,960 with lower expression in wild plants; and 5,441 in leaves, including 2,469 with higher and 2,972 with lower expression in wild plants (**Supplementary Table S7**). Because some unigenes may have been differentially expressed in more than one tissue, these tissue-specific counts were not summed to represent the total number of unique differentially expressed unigenes. A total of 65,693 unigenes were assigned GO annotations, comprising 343,955 gene-GO term assignments (**Supplementary Table S8**).

GO analysis revealed tissue-dependent functional differences between the wild and cultivated populations (**Figures 4A, C, E**). In roots, the enriched terms were mainly associated with intracellular structures and basic cellular functions. Cellular-component terms included protein-containing complexes, intracellular organelles, and the nucleolus, whereas molecular-function and biological-process terms were related to binding, catalytic and transporter activities, intracellular localization, and the regulation of cellular respiration. These results indicate that the root DEGs were broadly associated with cellular organization, transport, and primary metabolic processes.

**Figure 4:**
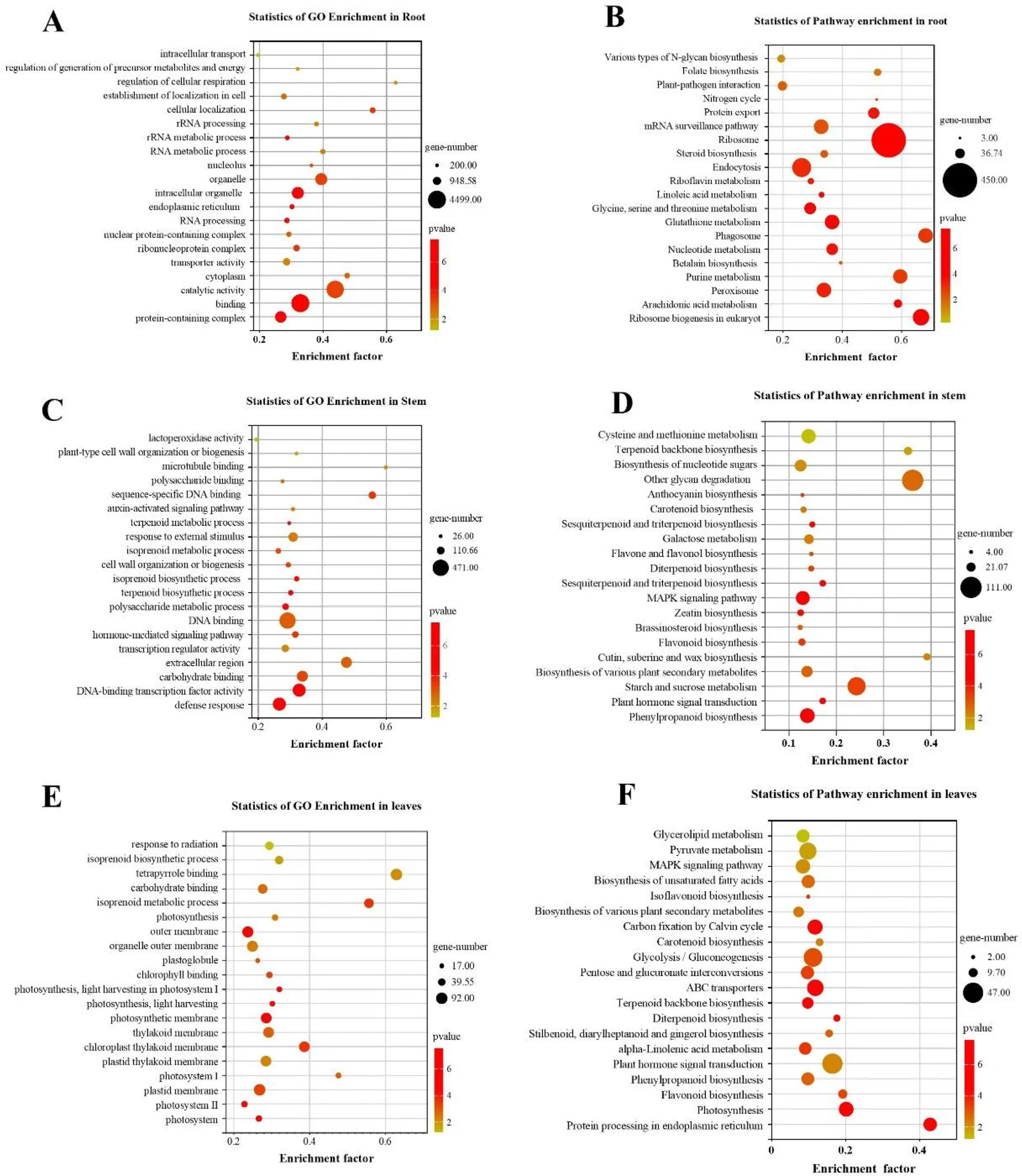
GO and KEGG analysis of the differentially expressed genes (DEGs); **(A, C, E)**. GO classification map of DEGs in root, rhizome, and leaf; **(B, D, F)**. 20 significantly enriched KEGG pathways in root, rhizome, and leaf.

In rhizomes, the enriched GO terms showed a stronger association with transcriptional regulation, environmental responses, and specialized metabolism. DNA-binding and transcription-factor activities were accompanied by defense responses, hormone-mediated signaling, responses to external stimuli, and auxin-activated signaling. The enrichment of polysaccharide metabolism, terpenoid biosynthesis, and isoprenoid biosynthesis further indicated that differences between the sampled wild and cultivated rhizomes involved both carbohydrate metabolism and pathways supplying precursors for specialized metabolites. However, pathway enrichment alone does not indicate the direction of metabolic change or the accumulation of the corresponding metabolites.

The leaf DEGs were primarily associated with photosynthetic structures and functions. Enriched cellular-component terms included the plastid membrane, thylakoid membrane, photosystem, and chloroplast thylakoid membrane. Consistent with these cellular locations, the associated biological processes and molecular functions included photosynthesis, light harvesting in photosystem I, chlorophyll binding, tetrapyrrole binding, and responses to radiation. Isoprenoid metabolic and biosynthetic processes were also represented, linking plastid-associated functions with the production of isoprenoid precursors.

KEGG enrichment analysis further distinguished the major functional patterns among the three tissues (**Figures 4B, D, F**). In roots, the enriched pathways included ribosome and peroxisome functions, together with riboflavin, nitrogen, and glutathione metabolism. Plant–pathogen interaction and steroid biosynthesis were also represented, indicating that the root comparison involved protein synthesis, nutrient and redox metabolism, and environmental-response pathways.

In rhizomes, the principal enriched pathways comprised starch and sucrose metabolism, galactose metabolism, zeatin biosynthesis, phenylpropanoid biosynthesis, and anthocyanin biosynthesis. This combination suggests that the rhizome DEGs were associated with carbon allocation, hormone metabolism, and specialized-metabolite biosynthesis. In leaves, enrichment was concentrated in photosynthesis, plant–pathogen interaction, and the MAPK signaling pathway, together with flavonoid, phenylpropanoid, terpenoid-backbone, carotenoid, diterpenoid, and α-linolenic acid metabolism. Thus, the leaf comparison involved both photosynthetic functions and several stress- and specialized-metabolism-related pathways. These enrichment results identify functional categories represented among the DEGs but do not, by themselves, demonstrate pathway activation or increased metabolite accumulation.

### 3.3 Comparison of terpenoid biosynthesis-related gene expression between wild and cultivated *Panax japonicus* var. *major*

*Panax japonicus* var. *major* (Zhuzishen), a rare and precious ginseng of the Araliaceae family, is rich in saponins. To date, 39 distinct saponins have been identified and characterized in the drug *Panacis Majoris Rhizoma*, including 11 panaxadiol saponins, 10 panaxatriol saponins, 14 oleanane saponins, 2 ocotillan saponins, and 2 kinds of saponin mother nucleus. The Chinese Pharmacopoeia states that the content of chikusetsu saponin IVa should be ≥ 3% as the standard for the quality evaluation of Zhuzishen.

The expression profiles of genes related to terpenoid biosynthesis were compared between wild and cultivated plants across roots, rhizomes, and leaves (**Supplementary Figure S1**). In plants, two metabolic branches relate to terpenoid production, namely the mevalonate (MVA) and the 2-C-methyl-D-erythritol 4-phosphate (MEP) pathways (Gupta et al., 2017). This study identified the genes encoding the enzymes required for the two pathways in the transcriptome data of wild and cultivated samples. In the MVA pathway, 10 enzymes (AACT, HMGS, HMGR, MVK, PMK, MVD, IDI, FDS, GDS, and TPS) that catalyze the biosynthesis of terpenoids were found in the transcriptome assembly. Furthermore, 7 enzymes unique to the MEP pathway were also identified, showing that transcripts assigned to both pathways were detected in *P. japonicus var. major* (**Figure 5**). The expression of DXS, which is involved in IPP and DMAPP biosynthesis through the MEP pathway, was higher in the sampled wild plants than in the cultivated plants, whereas no significant difference was detected in DXR expression (**Supplementary Figure S1**; **Supplementary Table S9**). The expression levels of the DEGs MCT, CMK, MDS, and HDS were also higher in the wild than in the cultivated plant, while HDR was an exception to this pattern. In addition, the expression levels of AACT, HMGS, HMGR, MVK, PMK, and MVD in the MVA pathway were higher in the wild than in the cultivated *P. japonicus* var. *major* (**Supplementary Figure S1**; **Supplementary Table S9**).

**Figure 5:**
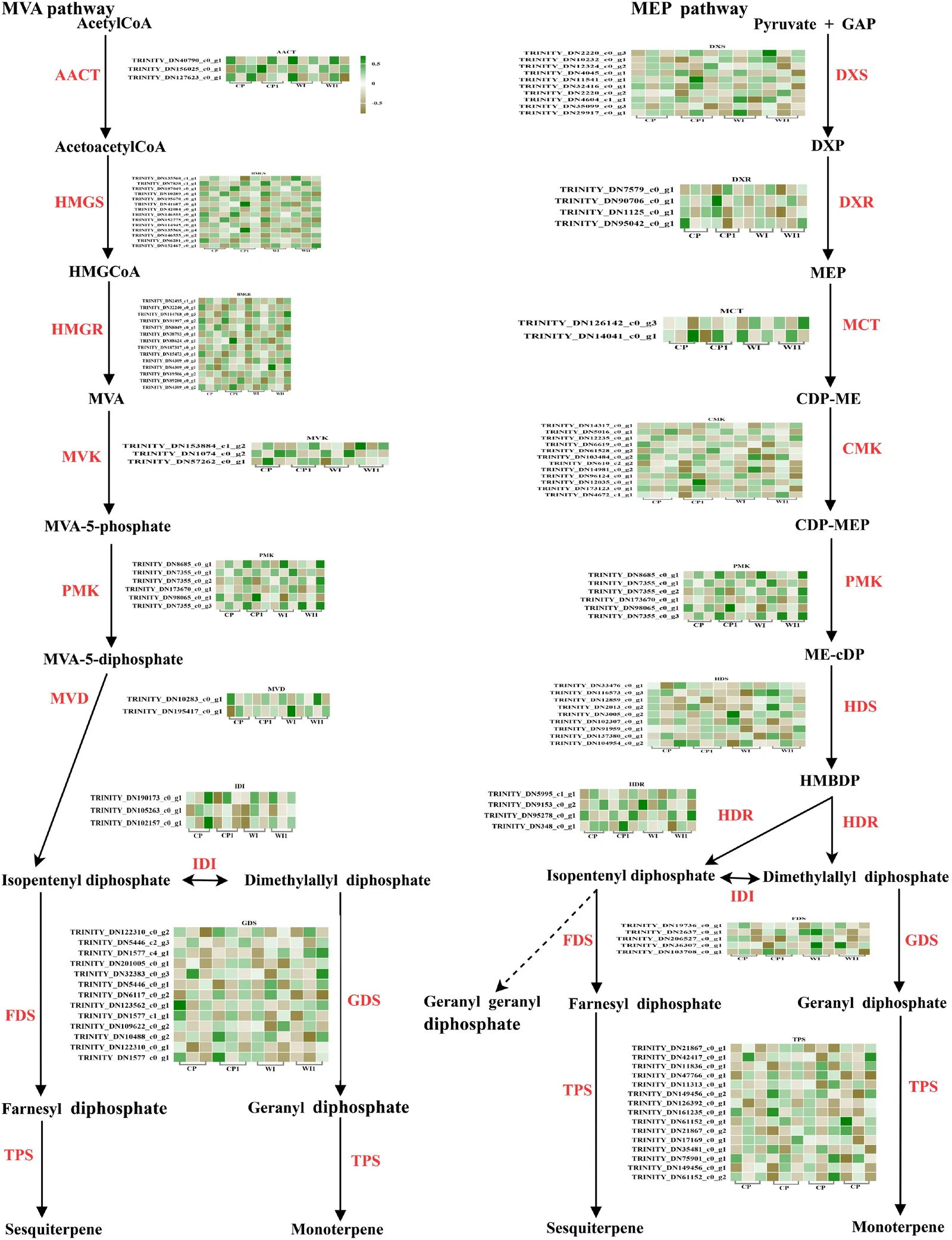
Terpenoid-biosynthesis pathways and transcript-abundance patterns in roots and rhizomes of wild and cultivated P. japonicus var. major. The 12 columns represent cultivated roots (CP; samples (E1–E3), cultivated rhizomes (CP1 in the original figure; samples (F1–F3), wild roots (WI; samples (A1–A3), and wild rhizomes (WI1 in the original figure; samples (B1–B3). In **Figure 5** only, CP1 and WI1 denote the rhizome transcriptome groups and do not refer to the additional microbiome sampling locations. Each tissue–population combination contained three independent biological replicates. Heat-map colors represent log2(TPM + 1)-transformed transcript abundance. Leaf expression profiles are presented separately in **Supplementary Figure S1**.

FDS, GDS, and TPS transcripts were more abundant in the sampled wild plants, whereas IDI showed the opposite pattern. AACT, HMGS, HMGR, DXS, and TPS also differed between the two sampled populations (**Supplementary Figure S1**; **Supplementary Table S9**). These results describe transcript-abundance differences only. Because terpenoid and saponin concentrations, enzyme activities, and medicinal efficacy were not measured, the data do not demonstrate increased metabolite accumulation or improved medicinal quality. They also do not establish that the observed expression differences were caused by the rhizosphere microbiome. A total of 16 terpenoid-biosynthesis genes, including AACT, HMGS, HMGR, DXS, DXR, HDS, HDR, FDS, GDS, TPS, MVK, PMK, MVD, IDI, MCT, and CMK, were selected to validate the RNA-seq results. The qRT-PCR results showed the same directions of up- or down-regulation as the RNA-seq data. Genes showing statistically significant expression differences in the RNA-seq analysis also showed corresponding significant differences in the qRT-PCR analysis (P < 0.05), demonstrating good consistency between the two methods (**Supplementary Figure S1**).

### 3.4 Comparative analysis of rhizosphere microbial communities in wild and cultivated *Panax japonicus* var. *major*

Bacterial 16S rRNA and fungal ITS sequencing was performed on 12 technical sequencing aliquots derived from four rhizosphere soil samples (WI, WI1, CP, and CP1; three technical aliquots per soil sample). These aliquots do not represent 12 independent biological samples. Accordingly, community composition and diversity are summarized descriptively, and no group-level inferential test treats the technical aliquots as independent replicates. After quality control and paired-read assembly, 625,613 bacterial 16S rRNA sequences and 638,482 fungal ITS sequences were retained. The descriptive OTU counts were 2,323 bacterial and 443 fungal OTUs in WI, 3,146 bacterial and 860 fungal OTUs in CP, 2,684 bacterial and 1,002 fungal OTUs in WI1, and 3,259 bacterial and 875 fungal OTUs in CP1 (**Supplementary Table S10**; **Supplementary Table S11**). Alpha diversity was evaluated using Chao1, Good's coverage, Shannon, and Simpson indices, together with the observed OTU counts (**Figures 6A–H**). Chao1 estimates richness, with larger values indicating greater estimated richness. Good’s coverage estimates sampling completeness, with values closer to 1 indicating more complete coverage. Shannon incorporates richness and evenness, with larger values indicating greater diversity. The Simpson index used here represents dominance; smaller values therefore indicate lower dominance and greater diversity.

**Figure 6:**
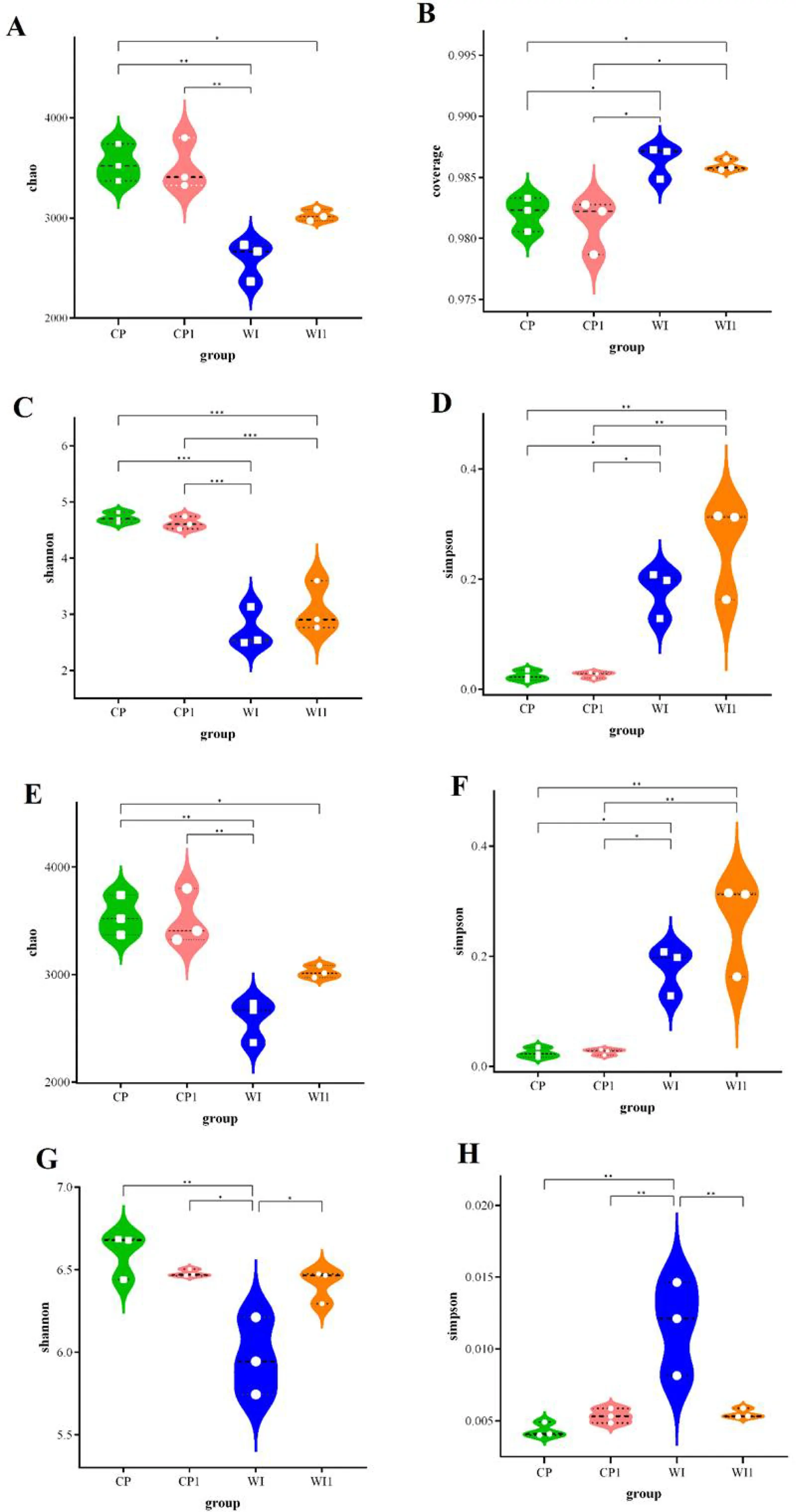
Analysis of richness (Chao1 index), diversity (Shannon and Simpson indexes), and the number of OTUs in samples of *Panax japonicus* var. *major*: **(A)** fungal Chao1 index; **(B)** fungal OTUs; **(C)** fungal Shannon index; **(D)** fungal Simpson index; **(E)** bacterial Chao1 index; **(F)** bacterial Shannon index; **(G)** bacterial Simpson index; **(H)** bacterial OTUs.

For fungi, the observed Chao1 values were higher in the cultivated samples, particularly CP, whereas Shannon and Simpson values did not show a consistent directional pattern (**Supplementary Table S12**; **Figure 6A**). Bacterial Chao1 values were also higher in the cultivated samples, particularly CP, while Shannon and Simpson values varied among the four soil samples (**Supplementary Table S13**; **Figure 6E**). Because each sampling group was represented by one independent soil sample, these differences are descriptive and no biological group-level significance is inferred.

Principal coordinate analysis (PCoA) based on Bray–Curtis dissimilarities (Fang et al., 2022) was performed on the rarefied OTU tables. For bacterial communities, PCoA1 and PCoA2 explained 47.70% and 17.70% of the variation, respectively, and the confidence ellipses showed substantial overlap among the four individual samples. For fungal communities, PCoA1 and PCoA2 explained 45.31% and 34.61% of the variation, respectively; a descriptive tendency was observed for the two wild-origin samples (WI and WI1) to be positioned on one side of PCoA1 and the two cultivated-origin samples (CP and CP1) on the other. However, because each sampling group comprised only one independent soil sample divided into technical aliquots, this PCoA pattern reflects variation among these four individual samples and cannot be interpreted as a population-level difference between wild and cultivated groups. PERMANOVA/Adonis, beta-dispersion testing, Kruskal–Wallis tests, ANOVA, LEfSe biomarker testing, and FDR-controlled group-level inference were not performed on the technical aliquots, because treating them as independent observations would constitute pseudoreplication.

At the phylum level, the rhizosphere soils of the four individual samples shared several dominant taxa, including Proteobacteria, Actinobacteriota, Acidobacteriota, Chloroflexi, Firmicutes, Bacteroidota, and Gemmatimonadota for bacteria, and Basidioycota, Ascomycota, Mortierellomycota, Rozellomycota, Mucoromycota, Olpidiomycota, Basidiobolomycota, and Glomeromycota for fungi (**Figures 7A, C**).

**Figure 7:**
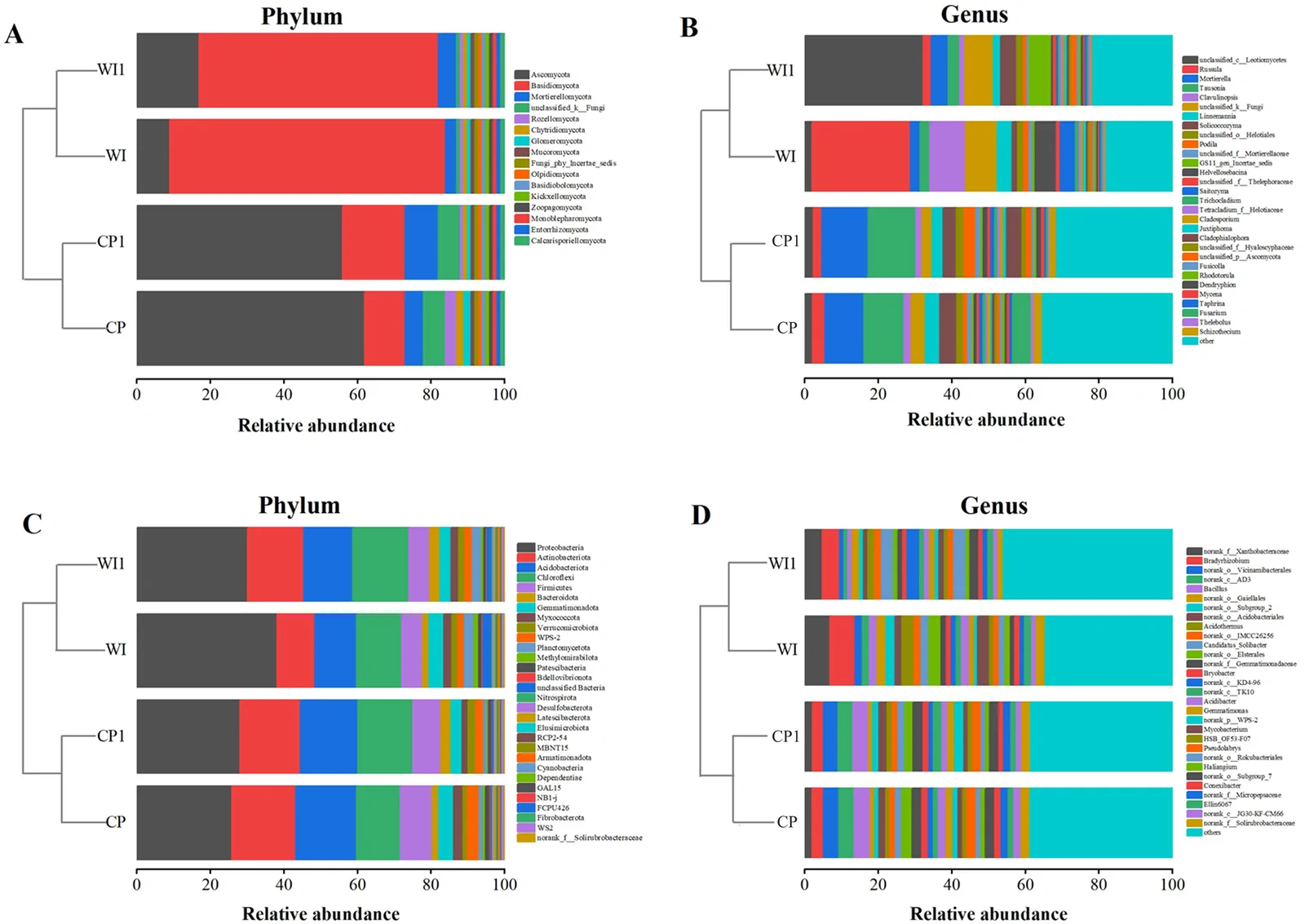
Relative abundances of microbial taxa in rhizosphere soil samples from CP, CP1, WI, and WI1: **(A)** fungal phyla; **(B)** fungal genera; **(C)** bacterial phyla; **(D)** bacterial genera.

At the genus level, bacterial and fungal relative-abundance profiles varied among the four rhizosphere soil samples. The 30 most abundant bacterial and fungal genera included in the descriptive comparisons are shown in **Figures 7B, D**. Because the within-group aliquots were technical replicates, the numbers previously described as significantly different genera have been removed.

LEfSe, Kruskal-Wallis, and ANOVA results are not used for biological group-level inference in the revised manuscript because each sampling group contained only one independent rhizosphere soil sample. Treating the three technical aliquots as independent replicates would constitute pseudoreplication. **Figure 8** therefore presents relative-abundance patterns only, without LDA-based biomarker claims, FDR-adjusted significance claims, or inferential P values.

**Figure 8:**
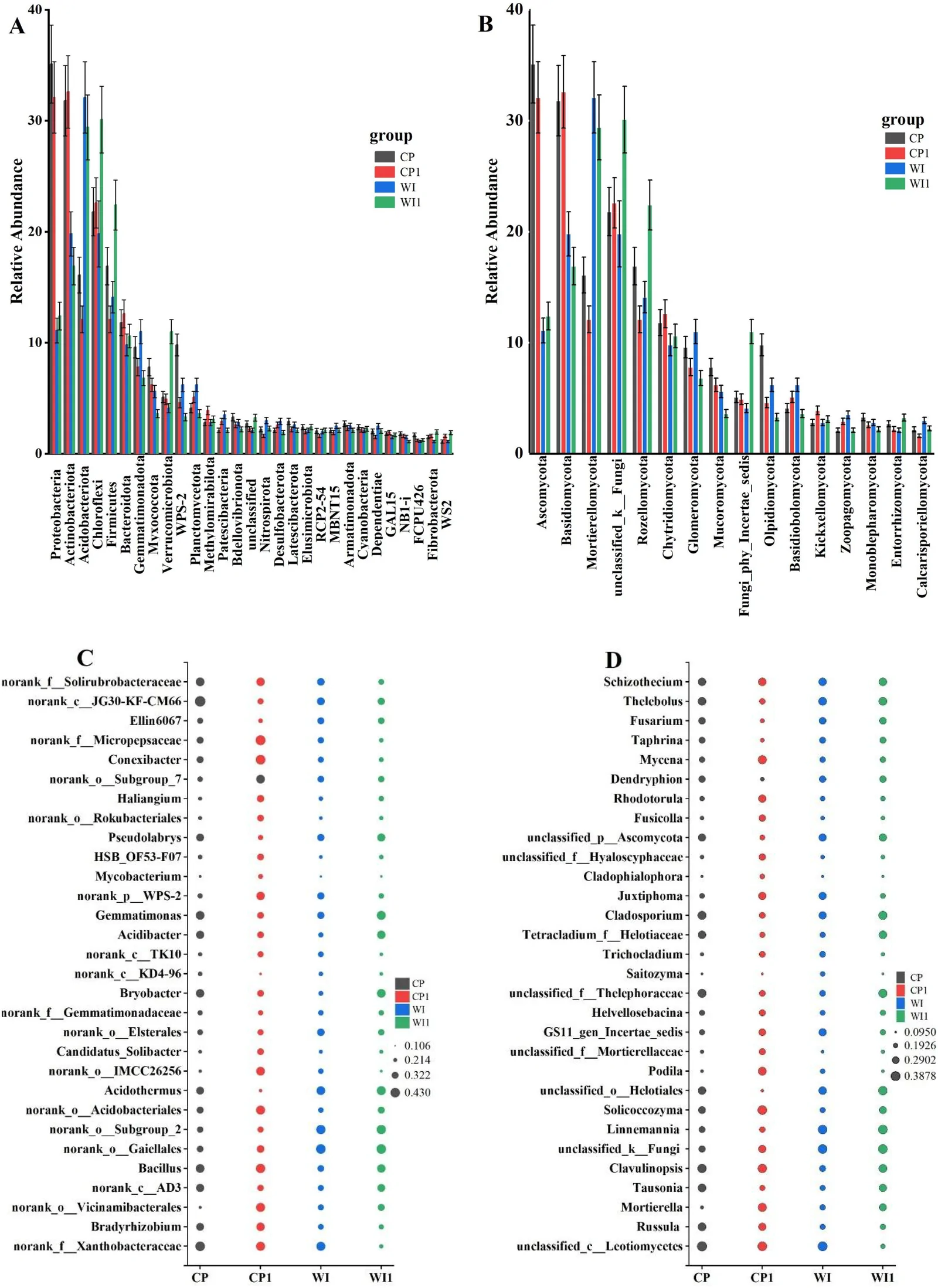
Descriptive relative-abundance patterns of bacterial and fungal taxa in cultivated (CP and CP1) and wild (WI and WI1) rhizosphere soil samples: **(A)** bacterial phyla; **(B)** fungal phyla; **(C)** bacterial genera; **(D)** fungal genera. No group-level biomarker inference is made from the technical replicates.

In the descriptive bacterial profiles, Xanthobacteraceae, Bradyrhizobium, Acidothermus, Mycobacterium, Granulicella, Flavobacterium, Beijerinckiaceae, and Puia had higher relative abundances in the sampled wild rhizosphere soils, whereas Novosphingobium, Hydrogenispora, Parablastomonas, Turicibacter, Romboutsia, Flavisolibacter, Paenibacillus, Blastococcus, Arthrobacter, Bacillus, Pseudonocardia, and Nitrospira were among the taxa with higher relative abundances in the sampled cultivated soils (**Figure 8**). These observations are not presented as statistically validated biomarkers.

In the descriptive fungal profiles, Russula, Clavulinopsis, Thelephoraceae, Helvellosebacina, Tylospora, Trichophaea, Xerocomus, Amphinema, Lactarius, Sebacina, Piloderma, and Inocybe had higher relative abundances in the sampled wild soils, whereas Tausonia, Solicoccozyma, Mortierella, Kernia, Podila, Dactylonectria, Juxtiphoma, Thelebolus, Pleotrichocladium, Tetracladium, Rhodotorula, and Monodictys were among the taxa with higher relative abundances in the sampled cultivated soils (**Figure 8**). These taxonomic patterns do not establish ecological function or statistical biomarkers.

### 3.5 Exploratory associations between gene expression and the rhizosphere microbial community of *Panax japonicus* var. *major*

WGCNA was used as an exploratory screening tool to identify candidate associations between gene-expression patterns and microbial abundance patterns. The resulting network contained candidate links between 1,284 fungal features from 16 phyla, 1,916 bacterial features from 30 phyla, and 14 terpenoid synthase-related genes (**Figures 9A–D**; **Supplementary Tables S14, S15**). Given the limited number of independent biological samples, the apparent modules and network edges should not be interpreted as stable or reproducible regulatory modules.

**Figure 9:**
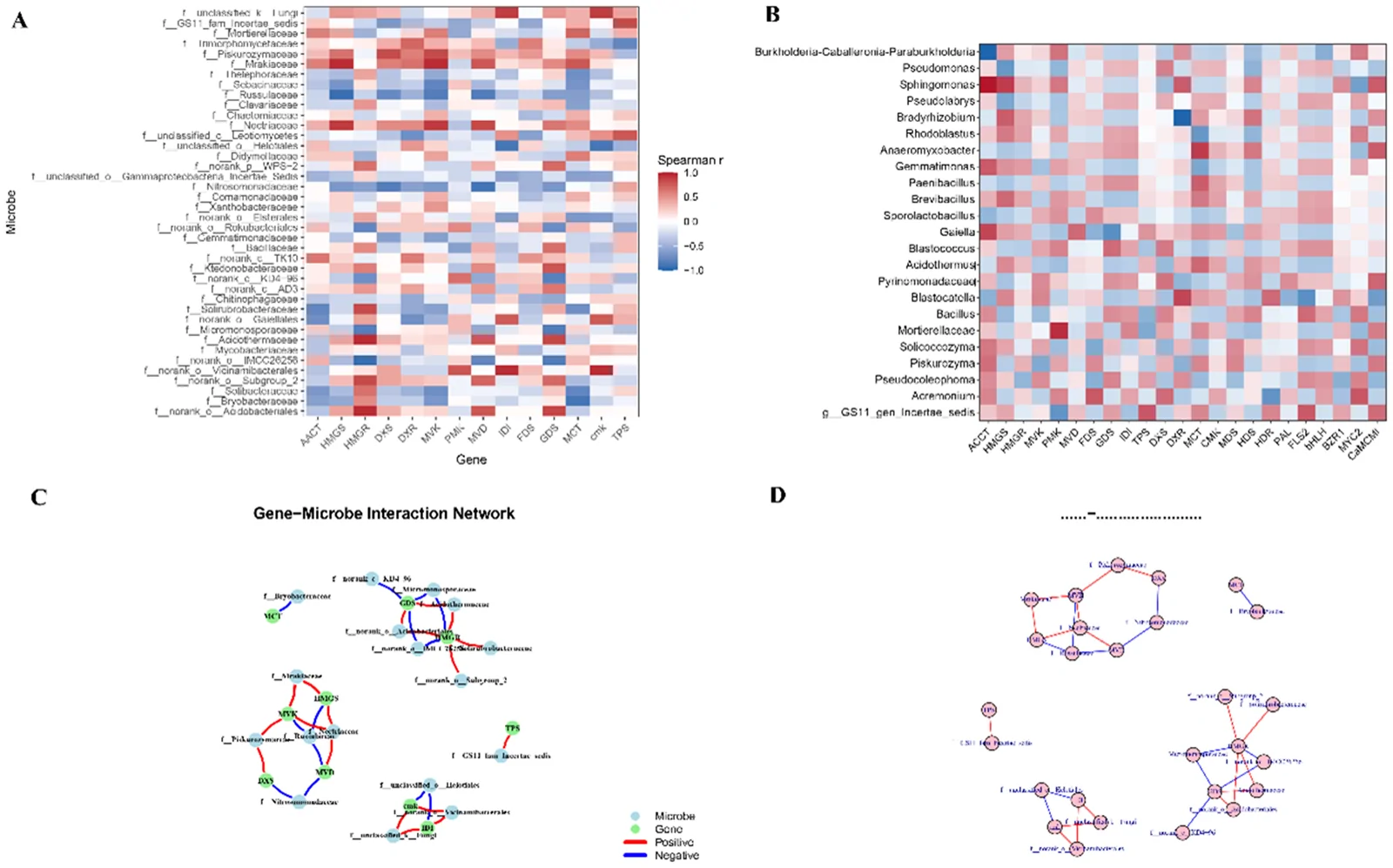
Exploratory co-occurrence patterns between microbial features and plant gene-expression traits. Red lines indicate positive correlations and blue lines indicate negative correlations. **(A)** Exploratory WGCNA network and candidate gene set–soil microbial taxon associations. Each row corresponds to a candidate microbial feature set, and each column corresponds to a gene-expression trait; **(B)** candidate gene set–soil microbial taxon associations; **(C)** candidate gene–soil microbial taxon associations; **(D)** selected candidate gene–microbial taxon associations. The limited sample size precludes interpretation of these patterns as stable or validated regulatory modules; they should be regarded as hypothesis-generating observations.

Positive correlations were observed between several microbial taxa and terpenoid biosynthesis-related genes. However, the 16S and ITS assignments were mainly at genus or family level and do not establish whether the detected organisms were plant growth-promoting, pathogenic, nitrogen-fixing, or capable of promoting terpene synthesis. No strain isolation or functional assay was conducted.

The exploratory cross-omics analysis identified candidate positive associations between terpenoid biosynthesis-related genes and several bacterial and fungal taxa (**Figure 9A**). These correlations are hypothesis-generating observations that require validation in larger, independently sampled populations. They do not establish stable network modules, microbial regulation of plant gene expression, or effects of plant genes on rhizosphere community composition.

## 4 Discussion

### 4.1 Tissue-dependent transcriptional differences between the sampled populations

The transcriptome comparisons revealed tissue-dependent differences between the sampled wild and cultivated populations. Root DEGs were mainly associated with cellular organization, transport, primary metabolism, and environmental-response pathways, whereas rhizome DEGs included hormone-signaling, carbohydrate-metabolism, and specialized-metabolism categories. Leaf DEGs were strongly associated with photosynthetic and plastid functions. Leaves were included as a non-medicinal, photosynthetic comparison tissue; their expression patterns were used to assess whether population-associated transcriptional differences extended beyond the medicinal rhizome and were not interpreted as direct evidence of rhizome chemical quality.

Several hormone-, MAPK-, and plant-pathogen-interaction-related genes showed different transcript abundance between the sampled populations. These patterns indicate differences in pathway-associated transcription, but the study did not measure hormone concentrations, reactive oxygen species, nitric oxide, pathogen pressure, or physiological stress tolerance. The results therefore do not establish pathway activation or greater stress resistance. Because the populations were collected from different field environments, the transcriptional patterns may reflect combined effects of population background, soil, microclimate, surrounding vegetation, and management rather than cultivation status alone.

### 4.2 Transcriptional differences in terpenoid biosynthesis-related pathways

Several genes in the MVA and MEP pathways had higher transcript abundance in the sampled wild plants. These expression patterns indicate differences in biosynthetic potential at the transcript level. They do not establish increased pathway flux, greater terpenoid or saponin accumulation, or improved medicinal quality because enzyme activities and metabolite concentrations were not measured. As illustrated by Huang et al. (2025), evaluation of medicinal chemical quality requires direct compound-specific measurements rather than transcriptional data alone.

Targeted LC-MS or HPLC quantification of major saponins is required to determine whether the observed expression differences correspond to differences in metabolite content. Such measurements should preferably be conducted in plants grown under controlled conditions so that chemical differences can be separated from sampling-site effects.

Accordingly, the present results identify population-associated differences in terpenoid biosynthesis-related gene expression. They do not demonstrate a domestication-driven reduction in secondary metabolite accumulation or medicinal quality.

### 4.3 Rhizosphere microbial patterns in the sampled wild and cultivated populations

The four rhizosphere soil samples differed in bacterial and fungal richness and community composition, but the technical replication structure does not support population-level significance testing. The PCoA plots therefore provide descriptive ordinations only: the bacterial samples overlapped substantially. The fungal samples showed only a descriptive tendency to separate along PCoA1, which does not imply a causal effect of cultivation.

Taxa with contrasting relative-abundance patterns were reported descriptively. Amplicon assignments at genus or family level do not establish whether the detected organisms were beneficial, pathogenic, nitrogen-fixing, plant growth-promoting, or involved in terpene metabolism. Functional conclusions require strain isolation, functional-gene evidence, and controlled inoculation experiments.

The cultivated and wild plants were sampled under different habitat and management settings. Shade netting was documented at the cultivated site, whereas soil physicochemical properties, detailed fertilizer and pesticide histories, vegetation effects, and microclimatic variables were not measured. Consequently, the observed microbial patterns cannot be attributed to a particular cultivation practice or environmental factor. Functions such as pathogenicity or plant growth promotion have been demonstrated for specific microbial strains under defined conditions (Nawaz and Bano, 2020), but they cannot be inferred from genus- or family-level amplicon assignments alone. No strain isolation, pathogenicity testing, or inoculation experiments were conducted in this study.

In this study, several bacterial taxa, including Burkholderia-Caballeronia-Paraburkholderia, Pyrinomonadaceae, Bradyrhizobium, Bacillus, Sporolactobacillus, Brevibacillus, Pseudolabrys, and Sphingomonas, showed contrasting relative-abundance patterns between the sampled wild and cultivated rhizosphere soils (**Supplementary Table S17**). These patterns describe taxonomic composition only. Although particular strains belonging to some of these taxa have been reported to influence plant growth or soil processes in other systems, the present amplicon data do not demonstrate those functions in *P. japonicus* var. *major*.

Previous studies have reported nitrogen fixation or nodulation by particular Bradyrhizobium strains and ecological associations involving other bacterial taxa (Fierer et al., 2007; Masuda et al., 2020). Other work has linked shifts in Bacillus, Pseudomonas, and fungal taxa to plant–soil feedback in continuously cropped Panax notoginseng (Luo et al., 2019). In the present study, Pyrinomonadaceae, Rhodoblastus, and Bradyrhizobium were relatively more abundant in the sampled wild rhizosphere soils, whereas Bacillus, Brevibacillus, Pseudolabrys, and Sphingomonas were relatively more abundant in the sampled cultivated soils. These observations cannot confirm nitrogen fixation, antagonism, stress resistance, or plant growth promotion because such functions are strain- and environment-dependent and were not tested here.

Previous studies have reported phosphate solubilization, siderophore or phytoregulator production, chitinolytic activity, and plant growth effects for particular Mortierellaceae isolates (Ozimek et al., 2018; Johnson et al., 2019; Li et al., 2020a). In our data, sequences assigned to Solicoccozyma and Mortierella alpina were relatively more abundant in cultivated rhizosphere soils, whereas Linnemannia and Mortierella elongata showed similar relative abundances between the sampled groups (**Supplementary Table S18**). These taxonomic assignments do not show that the detected fungi possessed the functions reported for previously studied isolates.

Sequences assigned to Fusarium, Bisifusarium, Phoma and Lectera were also detected and showed different relative-abundance patterns between the sampled groups. Although pathogenic strains occur within some of these genera, genus-level ITS assignments do not establish pathogenicity. No fungal isolates, pathogenicity tests, disease-incidence measurements, or inoculation experiments were conducted; therefore, the detected taxa cannot be classified as pathogens of *P. japonicus* var. *major* on the basis of the present data.

Overall, the results show differences in the relative abundance of bacterial and fungal taxa between the sampled wild and cultivated rhizosphere soils. They do not demonstrate that plant growth-promoting microorganisms were enriched in wild plants or that pathogenic microorganisms were enriched in cultivated plants. Functional interpretation will require strain-level identification together with functional gene, isolation, or experimental validation.

### 4.4 Research limitations and future validation

The field design cannot separate population or cultivation-related differences from the effects of soil, altitude, vegetation, microclimate, and management. The microbiome measurements were technical replicates derived from one soil sample per sampling group, and the WGCNA and cross-omics networks must therefore be regarded as exploratory. By contrast, previous studies on Panax rhizosphere microbiomes with adequate biological replication (e.g., Fang et al., 2022, with 5–7 independent replicates per group) were able to perform formal multivariate tests such as PERMANOVA and identify statistically robust differences among cultivation types. The present results should therefore be interpreted as descriptive observations from the four individual samples collected, generating hypotheses for future testing rather than establishing population-level differences. Genus- and family-level amplicon assignments do not establish microbial function, and the absence of terpenoid and saponin measurements prevents conclusions about chemical quality. These limitations are consistent with broader concerns about environmental confounding and incomplete gene-metabolite-function evidence chains in studies of medicinal plants (Suo et al., 2026; Guo et al., 2025).

Future work should use independently replicated common-garden experiments with standardized soil and management conditions. Soil physicochemical properties, vegetation, fertilizer and pesticide inputs, and microclimate should be measured concurrently. Targeted LC-MS or HPLC analysis, larger independent sample sets, microbial isolation, and controlled inoculation or synthetic-community experiments are needed to test the candidate associations. Furthermore, spatial metabolomics and *in situ* metabolite imaging could be applied to resolve the tissue-specific distribution of saponins and pathway intermediates, particularly in the medicinal rhizome, under controlled conditions (Zhu, 2025).

## Data Availability

The raw bacterial 16S rRNA and fungal ITS sequencing data generated in this study have been deposited in the NCBI Sequence Read Archive under BioProject accession number PRJNA1530771. The raw transcriptome sequencing data supporting the conclusions of this article are available in the NCBI Sequence Read Archive under BioProject accession number PRJNA1531093.
